# Commercialisation fears and preferred forms of governance: a mixed methods investigation to identify a trusted Australian genomics repository

**DOI:** 10.3389/fpubh.2024.1508261

**Published:** 2024-12-13

**Authors:** Brad Elphinstone, Jarrod Walshe, Dianne Nicol, Mark Taylor

**Affiliations:** ^1^Department of Psychological Sciences, Swinburne University of Technology, Hawthorn, VIC, Australia; ^2^Faculty of Law, University of Tasmania, Hobart, TAS, Australia; ^3^Melbourne Law School, The University of Melbourne, Parkville, VIC, Australia

**Keywords:** biobank, genomic, governance, trust, commercialization

## Abstract

This study aimed to identify operating conditions and governance mechanisms that would help to facilitate trust in, and willingness to donate to, a hypothetical Australian national genomic repository for health research where commercial use of data is permitted. Semi-structured telephone interviews with members of the Australian public (*N* = 39) clarified perceived risks and preferred repository conditions. These insights were subsequently tested experimentally in a national sample (*N* = 1,117). Contrary to what was expected based on the interviews, when certain baseline operating conditions were included (e.g., public management, data access committee to ensure data is restricted to human health research), none of the additional tested governance mechanisms (e.g., financial penalties for misuse) increased trust or donation willingness. Thus, providing suitable baseline conditions are in place, a feasible Australian genomic repository may not require external oversight or new legislation to optimize recruitment, even if commercial users are anticipated.

## Introduction

High-throughput, low-cost genome sequencing is driving the field of precision medicine, with repositories of human genomic data expanding in both number and scale (1). These genomic repositories provide biomedical researchers access to organized, high quality genomic data. Success is reliant on public willingness to donate genomic and relevant clinical data. As the sector grows, it is crucial to implement frameworks for governance of repository data that balances needs of different stakeholders (2). In this context, stakeholders include donors and their families, data generators, data users, developers of products and services arising from the use of data, clinicians, patients, and the public at large. The core tension repository organizations face lies in maximizing data value and the potential for scientific discoveries via broad data access, sharing, and use conditions whilst simultaneously managing risks and upholding public expectations (3, 4).

It has been well established that commercial involvement in genomic health research reduces public willingness to share personal genomic data compared to when data will be used only by academic or not-for-profit medical researchers (5–9). This is due to a range of concerns, such as concerns about privacy; unethical use of genomic data; that users of data (e.g., pharmaceutical companies) may engage in unfair profiteering from a resource comprising altruistic donations; and that donors may be discriminated against by law enforcement, employers or insurance companies [e.g., Vidgen et al. (8), Critchley et al. (10), Nicol et al. (11); see Walshe et al. (12) for a review]. Concerns regarding discrimination are particularly valid in the Australian context, which is of relevance to the current study, given that some Australians who have undergone genetic testing have been denied access to life insurance, even in the absence of disease symptoms (13).

Despite provoking public concerns, it is broadly acknowledged that large-scale public population genomic data repositories will at least partly rely on commercial arrangements to cover running costs and ensure ongoing financial sustainability (2). To ensure that public participation can be maintained despite commercial extraction of value from population genomic data, further public consultation is paramount. To date, research has principally focussed on identifying sources of concern around commercialization. Comparatively little research has explored repository governance mechanisms that may help temper these concerns, and thus sustain the public trust upon which these initiatives depend (12).

This paper is part of a larger Australian project [see Elphinstone et al. (14)] exploring community attitudes toward genomic data sharing and how public trust in a national genomic data repository can be preserved when shared data is available for commercial use. Australia is yet to develop a national genomic repository. The Genomic Health Futures Mission was created in 2019 with the intent to invest $500.1 million from the Medical Research Future Fund toward genomic medical research, with a stated aim of supporting the development of a national clinical and genomic data repository. This is the context from which the current study was formalized and funded.

Elphinstone et al. (14) conducted a nationally representative telephone survey (*N* = 1,000). The sample was then categorized using Latent Class Analysis (LCA) which identifies groupings (i.e., classes) of participants based on similar response patterns across survey items. Four classes were identified based on item responses regarding trust in, and willingness to donate to, repositories in which management is by Australian or overseas public (e.g., universities) or commercial (e.g., biotechnology) organizations, and that data could be accessed by public and/or commercial organizations. These classes are summarized in Table 1.

**Table 1:** Summary of the four classes identified by Elphinstone et al. (14) in a nationally representative sample of Australians.

**Class**	**Description**
Unsupportive	Distrustful of genomic repositories regardless of commercial involvement and unwilling to donate
Moderately supportive	Considers a national repository to be important and generally willing to donate to repositories with involvement by public institutions (i.e., hospitals, universities), but are uncomfortable with government or commercial involvement
Supportive	Considers a national repository to be important and generally willing to donate to repositories with involvement by public institutions (i.e., hospitals, universities), but are uncomfortable with commercial involvement
Highly supportive	Considers a national repository to be important and is willing to donate regardless of who manages the repository or has access to data

Elphinstone et al. (14) observed differences in perceived risks and support for certain governance mechanisms between the four classes. Highly Supportive respondents perceived lower risk of data misuse than members of the Supportive and Moderately Supportive groups. However, Moderately Supportive participants were statistically significantly more concerned about insurance companies gaining access to data than Supportive respondents. There were areas of general agreement across these three classes, such as the implementation of policies that ensure data remains anonymous and confidential. In contrast, no form of protection or governance mechanism enhanced trust and willingness to donate for Unsupportive participants.

The current study has two aims. The first is to qualitatively further investigate the perceived risks and preferred governance mechanisms held by participants across each of the four classes. Categorizing responses across these classes, rather than assuming the sample is homogenous, may assist in clarifying the extent to which certain views are reflective of the broader population. For example, dominant views within a sample would differ greatly if most in the sample identified as Unsupportive compared to another sample with a predominance of Highly Supportive respondents. Secondly, the implementation of governance mechanisms that have widespread support will be experimentally tested in a separate nationally representative sample to see if they enhance trust in, and willingness to donate to, a hypothetical national repository.

## Study 1—method

### Participants

Thirty-nine participants (18 men, 21 women) recruited from an earlier computer assisted telephone interview (CATI) survey [see Elphinstone et al. (14)] consented to a follow-up interview. All participants but two identified their cultural background as White Australian, aged from 26 to 83 (*M* = 52.74, *SD* = 12.92).

### Materials and procedure

One-hour semi-structured interviews (see Supplementary Appendix A for the interview guide) were conducted via telephone by two of the authors (JW and DN), with interviewees receiving an AU$50 gift card in recognition of their time. Interviewees were first called or emailed by JW to arrange a time for the interview and were then called directly by the researcher conducting the interview. Interviews commenced by explaining the role of a national genomic repository and how data would be used for health research. Respondents were asked about their knowledge of genomic health research and support for a national repository. This was followed with questions about repository management; consent; who should be allowed access to the data and under what conditions; restrictions on commercial use of the data; data privacy and protections for donors; and perceived risks. Interviews concluded by asking respondents to self-identify with one of four classes (see Table 1) identified by Elphinstone et al. (14).

### Data analysis plan

Reflexive Thematic Analysis (RTA) (15) was used. Themes were identified using an inductive approach and coded at a semantic level for each question based on the language participants used. Consideration was given to latent themes given participants' lack of experience with the topic. Interviews were conducted by JW and DN. Audio recordings were transcribed using artificial intelligence software (Otter.ai) and corrected for accuracy by JW. Utilizing the six-step approach of RTA, JW, and BE independently read the transcripts, developed initial codes, identified relevant themes, which were then reviewed and refined. The identified themes were those that reflected a consistent, patterned response or meaning. The analysis of each interviewee's responses was completed without any consideration of the class they self-identified with. Class membership was only used to compile the findings.

## Results

### Self-identified classes

Sixteen interviewees (41%) classified themselves into the Highly Supportive class, eleven (28%) as Supportive, eight (21%) as Moderately Supportive, and four (10%) as Unsupportive. One-third (*n* = 13; 33%) of interviewees self-identified with the group they were algorithmically allocated to by Elphinstone et al. (14). Toward a trusted genomics repository: Identifying commercialization fears and preferred forms of governance across segments of the community. *Public Understanding of Science* (in press). Many of the misclassified respondents (42%) self-identified with a class close to the one they had originally been allocated to (e.g., identifying as Highly Supportive but initially classified as Supportive). Inconsistencies may have partly occurred due to asking respondents to self-identify with a class at interview completion. For example, two further respondents (5%) described reconsidering their views since participating in the earlier survey.

### Knowledge of genomic research

Across each class, respondents generally had a vague understanding of genomic research, in some cases requiring further prompting and clarification of concepts to provide a response:

“*Having people's DNA and looking through the strands… and matching things with other things to see if you can pick up bits in it that may cause disease and that sort of stuff.”*—Woman, 54, Moderately Supportive

The Supportive and Highly Supportive classes had a higher concentration of respondents with quite sophisticated lay knowledge (“*…it's the identification of people's DNA, and how that might interact with health issues. Or extrapolating some of the information from the genomic data to formulate cures or treatments for other diseases”*, Man, 50, Highly Supportive). Several Highly Supportive group members had backgrounds in or adjacent to research; one was an academic, two worked in fields requiring oversight of company data, another had donated to genomic research, with another two participating in medical trials.

### Support for a national repository and willingness to donate

Most interviewees (*n* = 36, 92%) strongly supported genomic health research. No Unsupportive interviewees were willing to donate to a national repository *(“No way…not over my dead body. And not with my dead body either!”*—Woman, 43, Unsupportive). Perceived risks included reidentification by insurance companies, concerns about transhumanism, or that the government would create “harmful vaccines.” In contrast, every Highly Supportive interviewee supported a national repository and expressed willingness to donate, often highlighting a desire to contribute to the “greater good” through assisting in medical breakthroughs.

The Moderately Supportive and Supportive groups were more nuanced. All members of the Supportive group supported a national repository. Conversely, half the Moderately Supportive group were supportive, with the remaining half harboring concerns that tempered their support (“*I think it's great, the idea of it, but I'm undecided because being hacked and people's DNA used for other things is a real risk.”*—Woman, 48, Moderately Supportive). Members of both classes shared similar perceived risks and felt they would increase with commercial involvement, with discrimination from insurance companies or employers commonly mentioned.

“*It might be difficult to get insured. There might be forms of discrimination in terms of employment, educational opportunities, access to government benefits. All these sorts of things could be unintended consequences.”* (Man, 63, Moderately Supportive)

However, whereas four of eight Moderately Supportive interviewees were concerned about genetic discrimination, only three of 11 Supportive respondents were similarly concerned. The remaining eight Supportive group members felt that risks were low providing data is deidentified and protected from misuse. This was reflected in interviewees' willingness to donate.

The Moderately Supportive group included one member who was completely willing to donate, with the remaining seven interviewees expressing trepidation and a contingent level of support depending on who managed or had access to data (“*I think I would, I guess, depending on who had control of the information”*—Woman, 58, Moderately Supportive). In comparison, eight (of 11) Supportive interviewees were willing to hypothetically donate their genomic data, two expressed contingent support, and one was opposed.

### Commercial involvement in a national genomic data repository

#### Preferred management

Moderately Supportive interviewees preferred public organizations, such as hospitals and universities to manage the repository, as they were viewed, in comparison to commercial organizations, to have “*less of a financial incentive to sell the information on”* (Woman, 49). Another theme across this group was discomfort about the commercialization of data (“*I just don't like the idea of [donated genomic data] becoming commercialized.”*—Woman, 48, Moderately Supportive). Moderately Supportive respondents were split on their least preferred form of management. Some expressed low trust in government whereas others primarily distrusted commercial organizations.

For the Supportive and Highly Supportive groups, public organizations such as government institutes, universities, and hospitals were the favored form of management. This preference was influenced by perceptions that government would focus on the ‘greater good'. There were also views that public organizations would be more accountable and careful with handling data (“*I don't believe that a private entity would be able to hold that information as securely as a public entity would with more oversight and rigor.”*—Woman, 44, Supportive). Accordingly, all Supportive and Highly Supportive interviewees nominated commercial organizations as their least preferred form of management. References were made to “*unscrupulous big business”* (Man, 61, Supportive), with overarching concerns that profits would outweigh the public good, along with unchecked profiteering (“*I'm not happy about them profiting… when I say a profit, I mean an obscene profit not a reasonable profit. Sure, it's okay to make some money on it. But not an obscene amount.”—*Man, 50, Highly Supportive).

#### Restrictions on data use and access

The general view across each class was that data access, “*…should be restricted to genuine researchers or companies that are developing medications.”*—(Woman, 78, Highly Supportive). However, subthemes emerged in line with the fears and general trust in science that appeared to underpin each group. Members of the Moderately Supportive group highlighted cloning, eugenics, and genetic manipulation as necessary restrictions on use. Members of the Supportive group tended to say that data should only be used for ethical human health research. This was also emphasized by the Highly Supportive group; although, four (of 16) respondents were happy for the repository to determine appropriate use.

##### Commercial access

Most respondents in the Moderately Supportive (five of eight interviewees) and Supportive (nine of 10) groups, and all Highly Supportive respondents, supported commercial access providing there was suitable oversight, and that commercial organizations acted transparently. Concerns about corporate profiteering were again prominent, particularly in relation to overcharging for healthcare outcomes:

“*There must be a proviso that if everything is successful, and that the end result is [commercial organizations] come up with a drug or a cure for something, then it's got to be made available to the general public and not for free, because they've got shareholders and all that. But it has to be at a reasonable cost.”* (Woman, 55, Highly Supportive).

Despite these concerns, interviewees pragmatically supported commercial access. All but one Highly Supportive respondent, and half of the Moderately Supportive and Supportive groups, highlighted that commercial access would contribute additional funding and resources to bring new cures and treatments to market.

##### International access

Except for members of the Unsupportive group, one Moderately Supportive respondent, and two from the Supportive group, interviewees supported access being granted to international organizations. Those opposed were specifically concerned about China and Russia gaining access, with three interviewees suggesting that foreign companies would not be subject to Australian laws and controls.

Support for international access was guided by viewing research as a global endeavor, and Australian researchers should reciprocally share data with those overseas. These views of several respondents were influenced by international efforts to develop COVID-19 vaccines; “*Countries worked together, and we got a vaccine within a few months rather than a few years. So I think [repository data]…should be made available outside of Australia”* (Woman, 58, Supportive). Highly Supportive respondents uniquely highlighted that international access would be required as pharmaceutical companies are largely multinational corporations.

### Operation of a national genomic repository

#### Consent

All Unsupportive interviewees opposed broad consent, with most preferring consent to be sought for each use of data. One Unsupportive respondent opposed all forms of consent. Conversely, all but one member of the Highly Supportive group supported broad consent. These respondents highlighted that seeking consent for each use of data would increase costs and researchers' administrative burden. When asked if commercial access to data would change their consent preferences, the consensus amongst this class was, “*It depends on the commercial companies. If they were medical companies or related in that field it wouldn't change my mind, I'd be happy for it”* (Man, 50, Highly Supportive).

The Moderately Supportive and Supportive groups held similar views. Approximately half of these respondents expressed contingent support for broad consent, with concerns about commercial involvement.

“*If they're using it for profiteering, I would say no. But if they're using it for research into advancing human health, I'd be all for that. Again, I'm very suspicious of industry, especially when their motivations are commercial rather than advancing human wellbeing.”* (Man, 54, Moderately Supportive)

The remaining interviewees preferred consenting for each use of their data, but acknowledged the burden created for donors (“*If there's heaps of projects and you're getting asked every week, then it's a bit annoying*”—Man, 35, Moderately Supportive) and researchers *(“…you have to contact all these hundreds of people, but how do you contact them now because people have changed addresses and moved different places?”*—Woman, 44, Supportive).

For half of the Moderately Supportive group and for three (of 11) Supportive respondents, commercial access would change their consent preferences, citing concerns such as access by insurance companies and corporate profiteering. The remaining Moderately Supportive respondents and three further Supportive respondents said their willingness to consent would decrease if there was a risk of data being used for research other than for human health. Like the Highly Supportive group, four Supportive members were not concerned about commercial access, highlighting the role of the repository in ensuring appropriate use (“*I imagine that the repository is going to have to have guidelines around that…So, I think that would be part of the repository's role to assess that and decide”—*Woman, 65, Supportive). However, six (of eight) Moderately Supportive and nine (of 11) Supportive respondents wanted the ability to withdraw their data from the repository if they felt it was being misused. In contrast, withdrawing data was only considered important by four (of 16) Highly Supportive interviewees.

#### Data access committee

All interviewees, except for a single Unsupportive respondent, supported having a data access committee (DAC) to oversee data access. A combination of internal and external expertise was generally preferable across each of the four groups:

“*Well, I don't think it should be all independent, all outside of the company, because they may not know the facility—they may not know exactly what's going on. But it shouldn't be all internal either because you do need outside people to look at it.”* (Woman, 49, Moderately Supportive)

Two respondents in the Moderately Supportive class and four Supportive respondents preferred an external DAC feeling that it would have “more transparency” (Woman, 54, Moderately Supportive). In contrast, three (of 16) Highly Supportive interviewees supported an internal DAC due to the perception that external bodies will have their own agendas, be more expensive, and that an internal committee will better understand relevant policy.

#### Data access charges and benefit sharing

##### Access fees and royalties

All interviewees supported the repository recovering costs by charging for data access. Except for three members of the Highly Supportive group who preferred a flat fee for all researchers, interviewees preferred a tiered fee structure whereby public researchers would pay less than commercial researchers. This was often due to seeing commercial organizations as profit-seeking entities and universities or hospitals as serving the “greater good” and having less funding.

Approximately half of respondents suggested that a portion of profits from commercially successful outcomes be returned to the repository through a royalty system. However, this was rarely seen as important if a requirement to pay an access fee was in place. Five Highly Supportive respondents opposed royalties if an access fee had already been paid (“*…I don't think that would be ethical. It's one or the other.”*—Woman, 43, Highly Supportive).

##### Payment to donors

All but one respondent felt that donors should not be compensated as donation is an altruistic act (“*There's no flow back to individuals, you give it out hopefully with the idea that it's going to help society as a whole and as a result, hopefully helping those nearest to you.”*—Man, 49, Supportive).

#### Data security

Many interviewees vaguely referred to some type of state-of-the-art IT security to ensure that data is protected. Three respondents (two Supportive, one Highly Supportive) noted that data should be stored offline to minimize the risk of hacking. All Unsupportive respondents said that nothing could be done to reassure them about data security. Six (out of 16) members of the Highly Supportive group stated that they would simply trust that the systems and processes of the repository would be sufficient. Half of the Moderately Supportive and Supportive groups felt that nothing could be done, citing hacks of Australian consumer data which occurred during the timeframe of the interviews:

“*You just have to look at the data breaches we've had recently with Optus and Medibank … I think in the world we live in, the more you try and protect [the data], the more you're asking people to have a go at trying to access anyway”* (Woman, 58, Moderately Supportive).

### Punishments for misuse of data

All respondents felt that misuse of the data should be punished severely, with no discernible differences across classes. Thirteen respondents suggested severe financial fines and the threat of jail (“*Well, for businesses, heavy fines, and for individuals jail sentences…”*—Man, 51, Highly Supportive). Eight respondents stated that misuse should be met with considerable fines and being banned from all future data access. Six considered large financial penalties alone to be sufficient. Another three were satisfied solely with bans on future access. Eight respondents were unsure but felt that penalties should be commensurate with the extent to which public trust had been breached.

## Study 1—discussion

The qualitative results added to those of Elphinstone et al. (14), providing further insight into operating conditions and governance mechanisms that may enhance public trust in, and willingness to donate to, a national Australian genomic repository. While some interviewees expressed reservations about donating their genomic data, there was broad support, with two-thirds of respondents (predominantly from the Supportive and Highly Supportive groups) indicating that they would share their genomic data if asked, a finding in line with previous Australian research (5). While it appeared that little could encourage Unsupportive respondents to donate, very little would discourage Highly Supportive respondents. Therefore, identifying governance mechanisms that could allay concerns of Moderately Supportive and Supportive respondents appears to be important.

### Commercial involvement in a national repository

There was consensus that a national genomic repository in Australia should not be managed by government or commercial companies, but by public organizations such as universities or hospitals. This aligns with commonly identified concerns about commercial involvement in biobanking and genomic data repositories (12). This was especially important for members of the Moderately Supportive and Supportive groups, with the former being particularly concerned about government and commercial involvement.

In line with previous findings, there were also concerns about data being on-sold to insurance companies (10, 16, 17). While this was more pronounced in the Moderately Supportive and Supportive groups, concerns about misuse and corporate profiteering were also evident amongst members of the Highly Supportive group. As with other studies, participants across all classes were concerned about commercial organizations making ‘unfair' or ‘unreasonable' profits from donated data (18, 19) and the public being overcharged for research outcomes (20). Accordingly, there was support for commercial organizations paying more than public organizations for access to repository data (11). However, in line with previous findings (6, 21) there was an awareness, particularly within the Supportive and Highly Supportive groups, that commercial data use may yield novel drugs or treatments that would not have been developed otherwise.

Notably, despite some interviewees being concerned that misuse of data sent offshore would exceed the reach of Australian laws, there was reasonable support for foreign companies accessing data providing that the access conditions of the repository were adhered to. This contrasts with previous findings that have highlighted serious public concerns about overseas commercial researchers accessing data (10, 11). Given that several interviewees referred to international efforts to develop COVID-19 vaccines, an unexpected outcome of the pandemic may have been a shift in public views on international research collaborations. Therefore, while previous research has indicated that commercial management and access to genomic data is of concern to the public, the current study indicated that this concern is not homogenous throughout the community, and the types of commercial arrangements are important.

### Data access and penalties for misuse

The use of a DAC to ensure data is only used for ethical human health research was widely supported. A mix of suitably qualified internal and external experts would likely be deemed suitable by most Australians supportive of a national repository. This aligns with Nicol et al. (11) who identified public support for independent and transparent biobank governance. The availability of legislation to punish misuse of data (i.e., financial penalties, bans from future access, imprisonment) also received widespread support. Therefore, reassuring potential donors that a DAC is in place and that misuse of data will be penalized may help to mitigate concerns (e.g., data being accessed by insurance companies) held by people typical of the Moderately Supportive and Supportive groups.

### Consent

It is important to consider whether the broad consent model that is being utilized elsewhere (22) would be supported in the context of an Australian genomics repository. Most Highly Supportive interviewees supported broad consent, although only around half of Moderately Supportive and Supportive interviewees supported this consent model. This support declined slightly with the prospect of commercial access, which has been observed previously (21, 23). In line with previous research (16, 21, 23), there was widespread support across Moderately Supportive and Supportive respondents for donors to be able to withdraw their data from the repository. Given that Australian National Health and Medical Research Council research ethics guidelines require participants to be able to withdraw from research at any time, this requirement would need to be incorporated into a broad consent model and made clear to potential donors.

### Access fees and royalties

Moderately Supportive and Supportive interviewees generally favored tiered access fees, with commercial organizations expected to pay more than researchers from universities and hospitals, which supports previous findings (11). A royalty system whereby a portion of profits from commercially successful outcomes are returned to the repository also garnered support, although notably not by some Highly Supportive respondents if an access fee was already being charged. However, respondents were rarely strongly opinionated about either approach. It seems unlikely that the implementation of one approach over the other would affect willingness to donate. Participants simply wanted the repository to at least be able to recover costs and to ensure that commercial organizations pay their fair share. The issue noted by respondents of pharmaceutical companies potentially overcharging for discoveries would likely exceed the scope of the repository to address but should be considered by regulators.

### Data security

The salience of data security concerns amongst respondents was increased due to the interviews occurring in the wake of two widely publicized hacks of Australian consumer data. Numerous interviewees expressed that these types of hacks cannot be prevented where data is stored online. While most participants had little expertise in data security, a common suggestion was the use of state-of-the-art methods to protect against hacking and the malicious use of data. Ultimately, legislators need to ensure that privacy laws can meet the challenges resulting from technological developments, with particular focus on mitigating risks of donors being re-identified as donor privacy is an established concern (16).

### Conclusion and aims for Study 2

Based on the findings in light of the extant literature, particularly Australian-based studies (5, 9, 10), we considered there to be certain operating conditions which should be the baseline for an Australian genomic repository. These include public management (i.e., hospital or university); data use restricted to human health research; implementation of broad consent with the ability to withdraw data at any time; offline data storage on an Australian-based server; and a DAC comprising internal and independent genomic experts.

Other operating conditions and governance mechanisms that emerged in Study 1 with the potential to influence trust and willingness to participate were the extent to which commercial researchers could access data, access fees and/or royalties to help the repository recover costs, and penalties for misuse of data such as fines or imprisonment. Therefore, the focus of Study 2 is to investigate the extent to which implementing these forms of governance may increase trust in, and willingness to donate to a hypothetical Australian genomic repository.

## Study 2—method

### Participants

Respondents from a Qualtrics participant pool (*N* = 2,018) completed a 10-min online survey. After removing respondents where page timings indicated that background information text had been skipped, the final sample comprised 1,117 respondents aged from 18 to 99 (*M* = 53.57, *SD* = 16.95), including 614 men, 498 women, and five people who do not identify with binary gender labels. According to Douglas et al. (24), the quality of responses provided by participants from the Qualtrics pool is at least comparable, if not better, than those provided by participants on similar platforms (e.g., MTurk, Prolific).

Most respondents (75%) reported their cultural identity as White Australian, followed by British (7.7%), Chinese (2.6%), Indian (1.8%), Italian (1.8%), and a range of other backgrounds. Respondents reported their highest educational attainment as a vocational diploma or qualification (34.6%), high school completion (20.3%), undergraduate degree (19.7%), incomplete high school (15.4%), or postgraduate degree (9.9%). Most respondents were employed full-time (32.7%), followed by retirees (27.9%), those working part-time or casually (19%), homemakers (6.4%), unable to work due to disability (5.2%), unemployed (5.6%), or unspecified (3.3%). The sample was largely non-religious (55.6%), with a further 17.5% attending a place of worship less than once per year. The sample reported a centrist political orientation (*M* = 5.06, *SD* = 2.04) on a scale from 0 (Left) to 10 (Right). In comparison to 2021 census data (25), the current sample is older and comprises a greater percentage of males and non-religious individuals than the Australian population.

### Procedure and materials

#### Vignette presentation

Participants received a token payment from Qualtrics in exchange for their time. The survey (see Supplementary Appendix B) presented participants with different combinations of governance mechanisms utilized by a hypothetical Australian national genomic repository. All participants were first presented with descriptions of genomic health research and the purpose of a national genomic data repository. Based on the governance mechanisms that received widespread support in Study 1 and were deemed practical to implement based on Australian law, the aforementioned baseline conditions for a hypothetical repository were presented to all participants (e.g., public management, broad consent with ability to withdraw data).

This information was followed by randomly presenting different combinations of conditions to each participant. The first statement was about the users of the data (i.e., public and commercial health researchers, or public health researchers only). The second statement related to data access fees (i.e., all researchers pay a flat fee for access, or tiered access fees whereby larger organizations pay more for access). The third statement related to royalties being collected on commercially successful discoveries, and this was randomly presented to half of participants. The final statement related to penalties for misuse of data. The number of penalties was expanded based on a review of relevant legislative frameworks. Participants were randomly displayed between zero and three of the following, distinguished according to the nature of the penalty and entity responsible for initiating enforcement action: serious financial penalties and/or criminal prosecution (regulator action); donors affected by misuse being able to sue the offending organization for financial compensation (consumer action); and offending organizations forced to delete accessed data and are banned from future access (repository action).

#### Trust, willingness to donate, and concern about the repository

Following the vignette information, statements (see Supplementary Appendix B) were presented to ascertain levels of trust in a national genomic repository (0 = Would not trust at all, 10 = Trust completely); willingness to donate linked genomic and health data to a national repository (0 = Not at all willing, 10 = Very willing); and perceived importance of the Australian Government creating a national genomic repository (0 = Not important at all, 10 = Extremely important).

This was followed by seven statements regarding potential areas of concern (e.g., “Knowing who will use the data”; 0 = Not concerned at all, 10 = Very concerned). A further 10 statements, assessed on the same 11-point scale, asked about concern related to researchers from various organizations using the data (e.g., “Researchers from universities using my data”). These items were included for use in a LCA. Due to the heterogeneous nature of attitudes toward genomic repository management and governance, we again felt it important to consider the views of different segments of the community. The survey concluded with demographic questions (e.g., age, gender identity), and a question regarding current knowledge about genomic health research (0 = No knowledge, 10 = Very knowledgeable).

### Data analysis plan

To identify classes within the sample, LCA was used. A range of indices were examined to determine the most appropriate number of classes: entropy, Akaike Information Criterion (AIC), Bayesian Information Criterion (BIC), and Vuong-Lo–Mendell–Rubin (VLMR) indices. Entropy is an omnibus index in which ideal values range from 0.80 to 1, lower scores are preferable for AIC and BIC, and VLMR examines if a model with a certain number of classes provides better fit than a model with fewer classes. There are no consensus cutoff values for AIC, BIC, and VLMR. Hence, Nylund-Gibson and Choi (26) recommend that researchers consider the indices holistically to determine a justifiable number of classes.

Following the identification of classes, one-way Analysis of Variance (ANOVA) was conducted on each of the demographic variables, and variables assessing attitudes toward the repository and perceived concerns to explore differences between each class. A significant *F* test indicates that there is a significant difference in mean scores between the classes, with Tukey's corrected *post hoc* analyses used to identify specifically which classes significant differ from each other. Finally, multiple regression analysis was used to examine the extent to which each manipulated condition (e.g., presence of a royalty system) significantly predicted trust in the hypothetical repository and willingness to donate genomic data linked with health data.

## Results

### Identification of classes

As shown in Table 2, the VLMR index indicated that a four-class solution provided a better fit than a three-class solution. This was not improved upon with five classes. Furthermore, there was minimal change in the AIC and BIC values between the four and five class solutions which further supported the selection of a four-class solution.

**Table 2:** LCA results in the current sample.

	**Number of classes**				
	**1**	**2**	**3**	**4**	**5**
AIC	57,724.00	50,544.70	47,873.52	46,670.50	46,081.70
BIC	57,824.36	50,700.27	48,084.30	46,936.48	46,402.88
Entropy	–	0.95	0.94	0.93	0.92
VLMR	–	7,201.30^***^	2,693.17^**^	1,225.02^**^	610.80
*n* in each class	1,117	519, 598	334, 415, 368	296, 240, 366, 215	213, 239, 260, 147, 258

^**^p < 0.01.

^***^p < 0.001.

**Figure 1:**
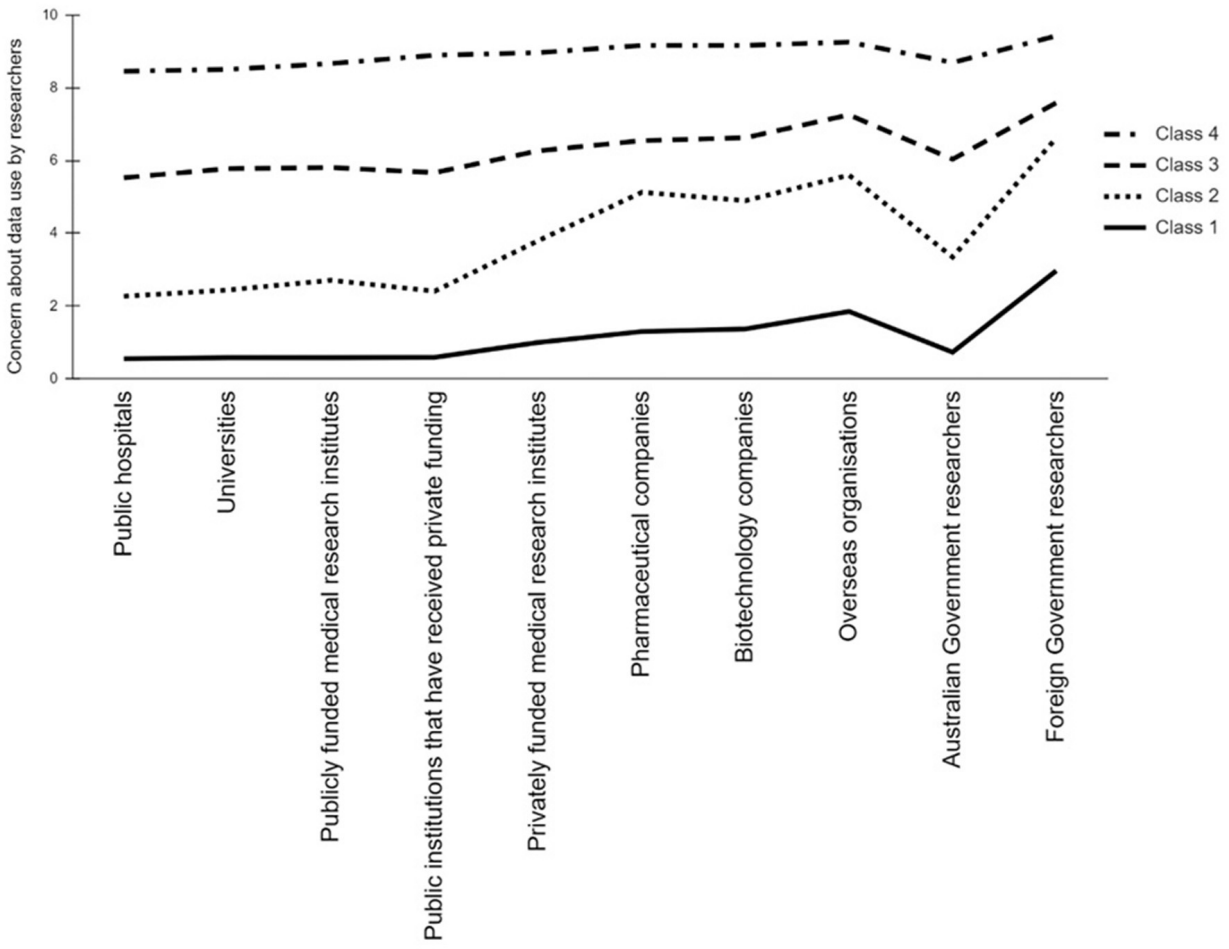
Mean scores for each class on items asking about perceived concern associated with data use by researchers from various organizations.

Mean scores for each class on the items used to derive the classes are shown in Figure 1. For ease of interpretation, we have used the same labels as the four classes described in Study 1, despite the classes being derived from different items than those used in Elphinstone et al. (14). Class 4 (*n* = 215; 19.25% of the sample) comprised respondents who were highly concerned about data being used by researchers from any organization, which aligns with the Unsupportive class. Conversely, Class 1 (*n* = 296; 26.50%) was marked by very low concern about researchers accessing data, thus aligning with the Highly Supportive class. Class 3 (*n* = 366, 32.77%) included respondents who scored slightly above the midpoint for each option and was labeled Moderately Supportive. Class 2 (*n* = 240; 21.50%) reported low concern about publicly funded researchers from hospitals, universities, or medical research institutes using the data, with moderate concerns for use by commercial researchers and those from foreign governments. This was labeled as a Supportive class.

#### Overview of the sample—attitudes toward the repository and concerns of misuse

The ANOVAs revealed significant differences (each *F* test *p* < 0.001) between the four classes on all variables except education and gender. Tukey corrected *post hoc* tests (see Table 3) revealed significant (*p* < 0.001) differences between the classes. The Supportive class was significantly older on average than the other three classes. The Moderately Supportive and Unsupportive classes reported greater religious attendance than the Supportive and Highly Supportive classes. While each class was politically centrist, the Unsupportive and Moderately Supportive classes reported a slight right-wing bias.

**Table 3:** Mean scores and significant differences for the overall sample and each identified class.

	**Overall sample**	**Class 1 (highly supportive) *M* (*SD*)**	**Class 2 (supportive) *M* (*SD*)**	**Class 3 (moderately supportive) *M* (*SD*)**	**Class 4 (unsupportive) *M* (*SD*)**
**Demographics**				
Age	53.57 (16.95)	53.65 (16.91)^a^	57.94 (16.15)^b^	50.85 (17.75)^a^	53.22 (15.50)^a^
Gender (1 = Male, 2 = Female)^*^	1.45 (0.50)	1.42 (0.49)^a^	1.39 (0.49)^a^	1.47 (0.50)^a^	1.51 (0.50)^a^
Education	2.89 (1.18)	2.90 (1.24)^a^	2.98 (1.16)^a^	2.83 (1.20)^a^	2.86 (1.10)^a^
Political orientation	5.06 (2.04)	4.83 (1.95)^a^	4.81 (2.16)^a^	5.20 (1.85)^ab^	5.41 (2.24)^b^
Religious attendance	2.09 (1.59)	1.83 (1.39)^a^	1.87 (1.45)^a^	2.27 (1.68)^b^	2.42 (1.73)^b^
**Attitudes toward the repository**				
Knowledge about genomic health research	3.07 (2.57)	3.21 (2.66)^a^	3.30 (2.48)^a^	3.17 (2.51)^a^	2.45 (2.62)^b^
Perceived importance of creating a national repository	7.18 (2.61)	8.83 (1.69)^a^	7.80 (1.97)^b^	6.87 (2.23)^c^	4.99 (3.16)^d^
Trust in an Australian repository	6.54 (2.47)	8.37 (1.66)^a^	7.13 (1.73)^b^	6.19 (2.00)^c^	4.27 (2.83)^d^
Willingness to donate	6.35 (3.04)	8.76 (1.85)^a^	7.33 (2.12)^b^	5.74 (2.53)^c^	3.33 (3.08)^d^
**Perceived concerns**				
Knowing who will use the data	5.92 (3.14)	2.50 (2.70)^a^	5.30 (2.64)^b^	6.90 (2.10)^c^	8.89 (1.41)^d^
Knowing who will profit from the data	5.92 (3.15)	2.55 (2.68)^a^	5.41 (2.75)^b^	6.84 (2.14)^c^	8.81 (1.56)^d^
That organizations will profit from the data	5.80 (3.17)	2.53 (2.82)^a^	5.29 (2.74)^b^	6.73 (2.12)^c^	8.55 (1.98)^d^
Data could be used for unethical research	6.40 (3.15)	3.86 (3.47)^a^	5.67 (2.91)^b^	7.12 (2.29)^c^	9.01 (1.43)^d^
Inability to opt-out of certain uses	5.98 (3.22)	2.79 (3.16)^a^	5.36 (2.87)^b^	6.93 (2.17)^c^	8.76 (1.49)^d^
Data used for non-medical research	6.53 (3.09)	3.88 (3.34)^a^	5.95 (2.98)^b^	7.27 (2.08)^c^	9.03 (1.46)^d^
Risk of experiencing negative consequences	5.32 (3.27)	2.42 (3.00)^a^	4.45 (2.90)^b^	6.12 (2.35)^c^	8.40 (1.93)^d^

Superscript letters indicate significant differences (p < 0.001) between classes.

^*^For this variable, individuals who identify with non-binary gender identities were not included in the analysis.

Each class on average reported low levels of knowledge about genomic research, with the Unsupportive class reporting the significantly lowest self-rated knowledge. Highly Supportive respondents reported the highest perceived importance, trust, and willingness to donate, followed in descending order by the Supportive, Moderately Supportive, and Unsupportive classes. Each class differed significantly, and this pattern was observed for all other variables. Thus, Highly Supportive respondents reported the lowest level of concern across all items through to the Unsupportive respondents who reported high levels of concern across all items.

#### Predictors of a trusted genomic repository

Due to the nature of the classes, the multiple regression analysis was conducted twice; first in the whole sample and then after excluding the Unsupportive class. Considering the Study 1 findings, Unsupportive respondents may be unwilling to donate under any conditions. Examining the three other classes in isolation may provide a clearer indication of preferred governance mechanisms by focussing on those who have at least a moderate level of trust and willingness to donate. Due to the design of the study and sampling, there was not enough statistical power to analyse each class separately.

The results in Table 4 indicated that in the entire sample, older respondents and those with tertiary qualifications reported higher levels of trust and greater willingness to donate. No additional governance mechanism was a significant predictor in the overall sample. After excluding the Unsupportive class and re-running the analysis, age significantly predicted trust and willingness, however, educational attainment no longer significantly predicted willingness to donate. The most notable emergent finding, contrary to expectations, was that when considered alongside the possible presence of donor or regulator-led penalties, the ability for the repository to impose penalties on those who misuse data was associated with reduced willingness to donate.

**Table 4:** Regression analysis results showing predictors of trust, willingness to donate, and perceived importance of creating a national repository.

	**All four classes**		**Excluding the unsupportive class**	
	**Trust**	**Donation willingness**	**Trust**	**Donation willingness**
*F*	2.97^***^	4.13^***^	6.13^***^	12.44^***^
**Covariates (unstandardised** β**)**				
Age	0.02^***^	0.03^**^	0.03^***^	0.04^***^
Gender	−0.12	−0.16	−0.13	−0.09
Tertiary education (No vs. Yes)	0.42^**^	0.46^*^	0.31^*^	0.26
Religious attendance (No vs. Yes)	−0.03	−0.20	−0.01	−0.18
Political orientation	−0.01	−0.04	−0.01	−0.05
**Governance mechanisms (unstandardised** β**)**				
Data users (public vs. public and commercial)	−0.09	−0.06	−0.14	−0.17
Fees (flat fee vs. tiered)	−0.03	0.14	−0.06	−0.03
Royalties (No vs. Yes)	0.02	0.07	0.01	0.10
Penalty (individual donors can sue)	−0.07	0.09	−0.18	−0.06
Penalty (repository can demand deletion of data and ban future access)	−0.17	−0.33	−0.24	−0.37^*^
Penalty (regulator can impose financial penalties and/or criminal prosecution)	0.08	0.02	0.05	−0.01

^*^ p < 0.05.

^**^ p < 0.01.

^***^ p < 0.001.

## Study 2 and overall discussion

The current study identified four subgroups, as have other Australian studies with similar sized samples [see Critchley et al., (32); Elphinstone et al. (14)]. Of note, in the current study and in Elphinstone et al. (14), < 20% of the sample (i.e., the Unsupportive class, 19.25% in the current sample) reported low trust and willingness to participate, with the majority reporting at least moderate trust and donation willingness. Thus, implementing governance mechanisms that can appeal to individuals typical of at least the Moderately Supportive class by helping to mitigate concerns about commercial access to and misuse of data, could be enough to garner majority public support.

### Predictors of repository trust and willingness to donate

Contrary to expectations, no proposed governance mechanism, beyond the baseline operating conditions described, contributed to increased trust or willingness to donate. This was unexpected given that many participants in Study 1 were clear about ensuring that the repository can recover costs from commercial use of data, and that misuse of data is met with severe penalties. Further, when the Unsupportive class was excluded from the analyses, the proposed option of repository action (i.e., users who misuse data are forced to delete it and are banned from future access) was associated with reduced donation willingness.

The unexpected findings accord with those of Briscoe et al. (3) where participants were randomly allocated to one of five conditions based on the type of organization requesting access to their genomic data: for-profit corporation, non-profit hospital system, university-run genomics and health research laboratory, global pharmaceutical company, or government research agency. The effect of various governance mechanisms (e.g., individuals can request their data be deleted any time) on willingness to donate were tested. The type of repository management did not affect the extent to which certain forms of governance affected willingness to donate. This contrasts with the expectation that donation willingness should be lower in the presence of commercial involvement (8, 10, 11).

The Study 2 findings and those of Briscoe et al. (3) indicate that people may, perhaps partly due to low knowledge about genomic research, be relying on heuristic judgments. Trust often functions as a heuristic to assist in simplifying complex decisions associated with biobanks when levels of risk are unknown (27). For example, trust in scientists has been associated with greater comfort with therapeutic cloning regardless of whether funding is public or private (20). In the current study, respondents across the Highly Supportive, Supportive, and Moderately Supportive groups appeared to have centrist or left-leaning political values, and Australians fitting that profile tend to be more trusting of scientists across a range of domains, such as vaccines and climate change (28).

Other demographic factors may have also accounted for why older respondents and those with university qualifications were more trusting and willing to donate to a hypothetical repository. For example, in other Australian studies with an overrepresentation of older respondents, trust in public compared to private biobanks was more pronounced amongst university educated respondents (5), and younger respondents were more likely to be classified as having reserved support of commercialization (32). Additionally, in a Swiss sample of people aged 60–89 years, Mählmann et al. (29) found strong support for personal genomic testing, often motivated by learning about one's disease risk.

Alternatively, the current findings may reflect that when participants have confidence that a repository will be run purely to support ethical human health research, other governance mechanisms, such as the imposition of penalties for misuse of data, become secondary concerns even with the possibility of commercial researchers gaining access to the data. For example, in Study 1 many respondents expressed pessimism about commercial organizations, believing them to be willing to act unethically in the pursuit of profit. Within this context, it is understandable that people would want commercial organizations to share profits in the form of royalties, and for punishments to be in place to mitigate against misuse. In Study 2, there was a baseline moderate-to-high level of trust and willingness to donate across the Highly Supportive, Supportive, and Moderately Supportive classes considering public management of the repository and use of a DAC. These operational policies may have provided sufficient assurance. Given that trust in scientists has been enduringly high in Australia between 2003 and 2020 (30), the Australian public may be willing to entrust access of their genomic data to a committee of scientific experts [see Kettis-Lindblad et al. (31) for a similar finding in a Swedish sample].

Further, perceptions of the DAC may have contributed to the unexpected finding that willingness to donate decreases when penalties for misuse are imposed. The need for these penalties may be seen to imply that the DAC is not doing its job properly, either because it is ineffective or overzealous in stipulating data usage. Given that the willingness to share genomic data is contextual, depending on the type of organization and intended use of data (9), the current findings may have been influenced by the context within which the DAC and penalties for misuse were proposed.

### Limitations and future directions for research

Study 1 inherited the sampling biases present in the CATI study from which the interviewees were recruited (14). Notably, the sample almost entirely included White Australians. While the sample in Study 2 was more diverse, it did not incorporate the views of First Nations Australians. Both samples were skewed toward older interviewees. Additionally, those who choose to participate in research, such as members of the Qualtrics participant pool used in Study 2, may not be representative of the average Australian. Thus, while participants in the current study appeared to be supportive of a DAC, this may not extend to diverse communities and/or those who are less interested in scientific research. Future research would also benefit from further exploring the expertise and backgrounds of members comprising DACs, as trust may be lower if members are connected to the commercial sector.

## Conclusion

The current study highlights the challenge in identifying what governance mechanisms might enhance public trust in, and willingness to donate to, a national genomic repository. This may be caused, in part, by low levels of knowledge and other demographic and/or contextual factors. However, considering previous research and in balance across both studies, the findings suggest that repository management should be independent of commercial interests, and assurances that all research and associated outcomes are done with integrity, transparency, and pursuit of the public good. The use of a DAC may assist in providing confidence that this will occur. As other governance mechanisms may not significantly affect trust or willingness to donate, an Australian national repository may not require substantial legislative changes (e.g., creation of specific criminal offenses for misusing data) or the establishment of external regulating bodies. Rather, providing that the operational conditions of the repository mitigate concerns about data being misused (e.g., on-sold to insurance companies), the repository may be considered trustworthy by most Australians. This should not deter the implementation of other measures by the repository that may enhance public trust in it or are desirable for other reasons. Although this study does not provide clear guidance on the hierarchy of options that should be considered, fees for data access and penalties for misuse should not be ruled out.

## Data Availability

The raw data supporting the conclusions of this article will be made available by the authors, without undue reservation.
