# Supplementary material for: Commercialisation fears and preferred forms of governance: a mixed methods investigation to identify a trusted Australian genomics repository
Source: Front Public Health. 2024 Dec 13;12:1508261. doi: 10.3389/fpubh.2024.1508261 (PMC11671527; doi:10.3389/fpubh.2024.1508261)
Supplement: Supplementary file 1 [file Data_Sheet_1.docx]

**Appendix A – Semi-structured interview guide**

**Towards a Trusted Genomics Repository: Tackling Commercialisation Fears**

**Telephone Interview**

**Introduction and oral consent statement**

Thank you for agreeing to participate in the next stage of the Trusted Genomics Repository research project. The purpose of this interview is to gain a better understanding of public views towards the establishment and governance of a national genomic data repository for health research with a focus on the controls that should or should not exist if a commercial company were allowed to use the data. There are no right or wrong answers, rather we are interested in your honestly held views.

Participation in this interview is completely voluntary and should take up to an hour depending on how much you might have to say. So that we have an accurate record of your words, the interview will be recorded and transcribed using a transcription service. After the transcribed data has been verified as accurate, the audio recording will be deleted.

Please note that all your responses will be de-identified and any data used in reports and publications resulting from this research will not be able to be used to identify you. You may decline to answer any questions and you may stop the interview at any time.

You also have the option to view a transcript of this interview, and if needed, to revise it before it is analysed.

To thank you for your time given in completing this interview, a $50 [Coles] e-gift card will be emailed to you at your preferred email address.

This research is being conducted as a joint project between Swinburne University of Technology, the University of Tasmania, and the University of Melbourne and is headed by Dr Brad Elphinstone. This research has been approved by Swinburne University’s Human Research Ethics Committee in line with the National Statement on Ethical Conduct in Human Research. If you have any concerns with the way this research is being conducted or would like to learn more about it, I can provide you with contact details of the Ethics Committee or the lead researcher. In the unlikely event that any of the questions asked causes you distress I can provide you with the contact details for Lifeline and Mental Health Online.

Do you have any questions?

Are you happy to begin?

**Background**

I’d like to begin by giving you some context about what we will be talking about today. As you may recall from our first survey, genomic health research will help to develop new treatments and drugs for cancers and other human diseases. This research relies on access to DNA information about lots of people. Using blood, saliva, or tissue samples, a person’s DNA is extracted in a laboratory, decoded into its basic letter code sequence, and saved as digital genomic data. The value of this data to researchers increases when linked to a person’s health history. A national genomic data repository would store data from hundreds of thousands or more Australians and make it available to researchers for current and not-yet-known projects. The data available to researchers would be de-identified, meaning it is assigned a code and would not contain details which could directly identify anybody. However, a small risk of re-identification exists because DNA is unique and will be linked to a person’s health history and other relevant clinical information.

The cost of establishing and operating a large-scale genomic repository is significant while the costs of translating genomic health research into new medical treatments can be enormous.

Governments around the world are encouraging collaboration between publicly funded genomic health initiatives and the private sector, where making the data widely available accelerates the rate of new discoveries and can help offset operating costs.

Today we’d like to hear your thoughts, both positive and negative, on the operation and governance of an Australian National Genomic Data Repository where commercial companies may have access to the data.

**For rapport**

**Q01.** To begin with, do you think that genomic research is a good idea?

**Follow up:**

What comes to mind you think of genomic research?

In your own words what would you describe genomic research to be?

**Q02**. Do you think it is important for Australia to establish a National Genomic Data repository for medical research?

**Probe**:

**If yes:**

What benefits do you think it would provide?

**If no or unsure:**

Can you expand on this?

**Q03**. If you were asked, do you think you would provide your linked genomic data to a National Repository?

**Probe**:

**If yes:**

What would be your motivation to provide your data?

**Follow up:**

What concerns would you have about providing your genomic data?

What could be done to make you more comfortable with the idea of providing your genomic data?

**If concerns are raised about providing linked data:**

Would you be willing to provide your genomic data if it was not linked to your health history data?

What specific concerns do you have about your genomic data being linked to your other health data?

**Management**

**Q04a**. Who would you trust most to manage the genomic repository?

If clarification needed or unsure: For example, the Government, a not-for-profit or independent foundation, a company, research institute, a hospital or university.

**Probe**:

Can you explain why you would most trust [entity] to manage the repository?

**Follow up:**

What are some things that [entity] might do that would decrease your trust in them?

**Q04b**. Who would you trust least (to manage the genomic repository)?

**Probe:**

Can you explain why you would least trust [entity] to manage the repository?

**Follow up:**

What are some things that [entity] might do that would increase your trust in them?

**Consent and information**

As already mentioned, the purpose of the repository would be to make the data available to researchers for current and future projects. Let’s now talk about consent.

**Q05.** What are your thoughts on the idea that you would be asked permission for each specific future use of your data? [opt-in]

And the idea that once a person consents to provide their data to the repository, they are consenting to all future uses of their data? [broad consent]

How about the idea that consent is automatic unless you specifically opt-out of a specific future use of your data? [broad consent with opt-out]

Finally, what are your thoughts on the idea that you could change your consent preference over time?

**Probe:**

Which model of consent would you prefer?

**Follow up:**

Would commercial companies having access to the data change the consent approach you’d like the repository to use? Why?

**Q06.** What information would you want to know about the projects that would be using your data?

**Follow up:**

Would you want to be informed of the intended benefits of the research before the project begins? Why?

What about being informed of the actual benefits of the research once the project is complete? Why?

**Probe:**

Is one of these more important than the other? Why?

How would you want the results of the research shared?

**Q07**. Is there anything you would not want your data to be used for?

**Probe:**

Why not?

**Access**

**Q08.** Do you think it is appropriate for the repository to charge for access to the data?

**Probe:**

Why / why not?

**Q09.** If the repository did charge for access, do you think access charges should be the same for all researchers or organisations?

**Follow up [if not brought up by participant]:**

Do you think that commercial companies should pay more for access than, for example, a public university or not-for-profit medical research institute?

**Probe:** Why / Why not?

**Q10.** Who do you think should be responsible for deciding who can access the data?

**Follow up:**

Do you think the repository should have an internal committee of repository staff and experts that decides access?

Or

Do you think the repository should have an external access committee made up of independent experts including community representatives that decides access?

**Probe:** Do you prefer one access committee idea over the other?

What do you think would be the benefit of this type of committee?

**Q10.1** What are your thoughts about allowing people to choose who can access their data?

**Probe:** How would you want that to work?

**Q11.** Do you think the data should be made available under a consistent set of rules for all organisations? Why / why not?

Or

Do you think that special rules should apply to certain organisations but not others? Why?

**Q12.** Do you think there should be restrictions placed on who can access the data?

**Probe**:

What restrictions and on whom / why not?

**Q13.** Thinking not about the user but the use, do you think there should be restrictions on how the data can be used? e.g. areas of research where you wouldn’t want your data to be used.

**Probe:**

What kind of use restrictions do you think should be in place? Why?

**Commercial use of the data**

**Q14.** Do you think it is fair that commercial companies be allowed to access the data?

**Probe:**

Why / why not?

**Follow up questions:**

Are there any circumstances in which you think it would be [fair/unfair] for a company to access the data?

**Probe:**

What if it led to treatments that would otherwise not be developed?

What if a proportion of commercial profits were redirected back to public health research?

**Q15.** Can you think of any benefits in allowing commercial companies access to the data?

**Follow up** [after time for reflection]:

What kinds of companies would provide benefits if they were allowed to access the data?

Does this affect your view that access is fair/unfair?

**Q16.** Do you think it is fair that universities or public research institutions be allowed to access the data?

**Probe:**

Why / why not?

**Q17.** Should universities or other public research institutions be allowed to profit from the data?

**Probe:**

How is this different from commercial companies profiting?

**Q18.** Do you think anything is owed to the repository if a significant commercial discovery is made using the repository’s data? To the participants who provided the data?

**Probe:**

What do you think should be owed and to whom? e.g. Financial returns? Profit / knowledge sharing? Reporting of results? Other?

**Q19.** If the data were to be made available to commercial companies, should this be restricted to Australian companies only?

**Probe:**

Why? Are there risks or limitations in restricting/allowing access to [Australian/foreign] companies?

**Data privacy and protections**

**Q20.** How do you think your data should be protected?

**Follow up:**

What would reassure you that your data is being protected?

**Q21.** How sensitive would you say your genomic data is? Do you think this data is more or less sensitive than other types of personal data?

**Follow up:**

Do you think different rules should apply to access to genomic data than health data? Why?

Should there be different rules/special rules for commercial access in relation to data privacy and protection? Why?

**Q22.** Do you think that de-identified genomic and health data should be protected by privacy legislation? Why?

**Q23.** Do you think there should be penalties for misusing the data? What penalties do you think should be in place?

**Risks**

**Q24.** Can you think of any risks that you might experience if you provided your linked genomic data to the repository?

**Q25.** Do you think your data is at greater risk if shared with commercial organisations?

**Probe:** Why / why not?

What type of risks? e.g. privacy breaches / unauthorized access or sharing / misuse of data

**Follow up:**

What do you think could be done to help lessen these risks?

What can commercial organisations do better to reduce these risks?

**Q26.** How much would the risk of re-identification worry you?

**Follow up [if yes]:**

Does this affect your willingness to participate?

**Q27.** Do you have concerns that your data could be accessed for reasons other than for medical research?

**Probe:**

If yes, who do you think might want to access the data? What harms do you think might come of this?

**Q28.** Would you be worried that rules to access the data would change in the future?

**Follow up [if yes]:**

What would reassure you that the rules wouldn’t be changed?

**Q29.** Previous research has indicated that there are broadly four classes of people in terms of comfort in providing their linked genomic information for health research to public and commercial entities. The four classes are:

1. people who are generally comfortable providing their data for public health research regardless of who will be using it
2. people who are not comfortable providing their data at all
3. people who are comfortable providing their data for public health research to universities, public institutions, and government to use but are less comfortable with commercial companies having access
4. people who are comfortable providing their data for public health research at universities and public institutions to use but are less comfortable with government or commercial companies having access

Which of these classes best describes you?

That’s the end of the questions. Do you have any final thoughts you would like to share?

Thank you very much for your time today, your contribution is greatly appreciated.

**Appendix B – Study 2 survey materials**

**BACKGROUND INFORMATION**

**Please read the following background information carefully**

**What is genomic health research?**

Genomic health research will help to develop new treatments and improve healthcare for cancers and other human diseases. This research relies on access to DNA information about lots of people.

Using a biological sample, a person's DNA is extracted in a laboratory, decoded into its basic letter code sequence, and this information is saved as digital genomic data. The value of this data to researchers increases when it is linked to a person's health history (e.g., a history of cancer or other serious health conditions).

Genomic health research looks at this information from thousands of people collectively to understand how certain genes contribute to illnesses and diseases. The aim of genomic health research is to develop cures or treatments, or help existing care and treatment to be earlier and better targeted.

**What is a National Genomic Data Repository?**

The Australian Government is interested in opening a National Genomic Data Repository. This would store data from hundreds of thousands or more Australians. The data would be made available to health researchers today, but also for projects in the future that have not been thought of yet but are intended to improve health and treatment outcomes.

The data available to researchers would be de-identified, meaning that it is assigned a code and it would not contain details which could directly identify anybody. However, your DNA information is unique. If a motivated person matched other genetic and personal data with the information in the repository, there is the possibility that they could re-identify someone.

The cost of establishing and operating a large-scale genomic repository is significant. Further, the costs of translating this type of research into new medical treatments can be enormous. Accordingly, governments around the world are encouraging collaboration between publicly funded genomic health initiatives and the private sector. Making the data available widely to as many parties as possible accelerates the rate of new discoveries and can help offset operating costs.

The aim of this study is to learn about your views on providing your genomic and health data to a National Genomic Data Repository and the legal controls the repository should be subject to before a commercial health research organisation is provided access.

**SCENARIO**

**Please carefully read and consider the following scenario about how the repository would operate:**

During a routine medical appointment, you are asked to contribute to health research by providing your **genomic and health history data** to the Australian National Genomic Data Repository.

**Management**

The repository would be **publicly managed** and receive partial funding by the Australian government.

**Purpose of the repository**

The data would be available **strictly for human health research** to improve medical care and the treatment of diseases and illnesses.

**Consent**

The repository would operate under a model of **broad consent**. This means that you would be asked to consent once for indefinite use of your data by health researchers. You would be able to **withdraw your consent** at any time and the repository would **delete your data**. This would stop any further use of your data.

**Data security**

The repository would store the genomic and linked health data **offline** on a server in Australia. The data would be made available to researchers in a **de-identified format** where all personally identifiable information has been removed.

**Users of the data**

***[1 of the following will be randomly displayed to survey respondents]***

1. The de-identified repository data would be available to both **public and commercial** health research organisations. Examples include universities, medical research institutes, pharmaceutical and biotechnology companies, and hospitals amongst others.

2. The de-identified repository data would be available to **public** health research organisations only. Examples include universities, medical research institutes and hospitals amongst others.

**Access to the data**

The repository would use a **Data Access Committee** to decide who can access the data. The access committee would be made up of both **internal and independent** genomic health research experts. Researchers wanting to use the repository would need to submit a **detailed plan** describing the aims and intended benefits of the research and how the data will be used. Applications from overseas researchers would be subject to additional scrutiny to ensure the research aligns with Australian ethical and legal standards.

**Access Fees**

***[1 of the following will be randomly displayed to survey respondents]***

1. The repository would charge an access fee to recover operating costs. **All researchers and organisations would pay the same flat fee**.

2. The repository would charge an access fee to recover operating costs. Access fees would be **tiered** and based on the number of employees in the research organisation where **larger organisations pay more** to access the data.

**Royalties**

***[This section will appear randomly to half the survey respondents]***

The repository **will collect royalties on commercially successful discoveries** made using its data. The royalties collected will be used to support the repository and to further fund public health research.

**Penalties**

**[*Participants will randomly see between 0 and 3 consequences for misuse of the data*]**

The data would be **protected by privacy legislation** and its use by research organisations would be subject to a **legally binding contract** with the repository.

If the research organisation **failed to meet their responsibilities in the handling or use of the data** the following consequences apply:

1. They would face **serious financial penalties and/or criminal prosecution** by the regulator.
2. They would be liable to **be sued for financial compensation** by individual repository participants.
3. They would have to **delete any data** they had received and be **banned from future access** by the repository.

**QUESTIONS**

Based on the information presented regarding how the National Genomic Data Repository would operate…

1. Overall, how much would you trust a Australian National Genomic Data Repository?
   [0 = Would not trust at all, 10 = Trust completely]
2. How willing would you be to donate your linked genomic and health data to an Australian National Genomic Data Repository for health research?
   [0 = Not at all willing, 10 = Very willing]
3. How important do you think it is for the Australian government to create an Australian National Genomic Data Repository for health research?
   [0 = Not important at all, 10 = Extremely important]
4. How concerned would you be about the following…
   [0 = Not concerned at all, 10 = Very concerned]

[Randomise]

- 1. Knowing exactly who was using my genomic data.
  2. Knowing the exact purpose my data was being used for.
  3. Knowing who would be benefitting from the use of my data.
  4. Knowing who would be profiting from the use of my data.
  5. That organisations would profit from the use of my data.
  6. That a future request to delete my data would **NOT** be acted upon.
  7. That my data would be used for research that was unethical.
  8. The inability to opt out of having my data accessed for certain purposes.
  9. That my data would be used for reasons other than medical research.
  10. That I would experience negative consequences by providing my data.
  11. Researchers from public hospitals using my data.
  12. Researchers from universities using my data.
  13. Researchers from publicly funded medical research institutes using my data.
  14. Researchers from public hospitals, universities, or medical research institutes that have received private funding using my data.
  15. Researchers from privately funded medical research institutes using my data.
  16. Researchers from commercial pharmaceutical companies using my data.
  17. Researchers from commercial biotechnology companies using my data.
  18. Researchers from overseas institutes or organisations using my data.

1. What, if anything, would increase your level of trust and willingness to donate your genomic and health data to an Australian National Genomic Data Repository?

[Open text box for participants to provide a qualitative response]

**Demographics**

1. What is your age in years? ______
2. What is your gender identity?
   [Woman / Man / Non-binary / I identify as ______ ]
3. What cultural background do you most identify with?
   [Australian / Chinese / English / German / Greek / Indian / Irish / Italian / Scottish / Vietnamese / Other: _______]

1. What is your current employment status?
   [Work full time / Work part time or casually / Home duties / Retired / Disability, Workcover, or other support payment (excluding JobSeeker) / Looking for work / Other: ________ / Prefer not to answer]
2. What is the highest level of education you have completed?

[Below Year 12, including Certificate I & II / Year 12 or equivalent / Certificate III, IV or Diploma / Undergraduate degree / Postgraduate degree]

1. Which State or Territory do you live in?
   [ACT / NSW / NT / QLD / SA / TAS / VIC / WA]

1. Apart from special religious ceremonies such as weddings and funerals, how often do you attend religious services?
   [Never / Less than once a year / At least once a year / Several times a year / At least once a month / At least once a week]
2. In political matters, people talk about the ‘left’ and the ‘right’. On a scale from 0 to 10, where 0 = Left and 10 = Right, where would you place your views generally speaking?
3. How would you rate your current knowledge about genomic health research?

[0 = No knowledge, 10 = Very knowledgeable]

1. Have you ever provided your genomic data for genomic health research?
   [Yes / No / Unsure / Prefer not to answer]
